# Pulmonary tuberculosis complicating post patent ductus arteriosus ligation recovery: a case report

**DOI:** 10.11604/pamj.2023.44.104.39062

**Published:** 2023-02-23

**Authors:** Uchenna Chinweokwu Onubogu, Martha Elizabeth Okorie

**Affiliations:** 1 Department of Paediatrics and Child Health, Rivers State University Teaching Hospital, Port Harcourt, Rivers State, Nigeria,; 2 Department of Paediatrics, Faculty of Clinical Sciences, Pamo University of Medical Sciences, Port Harcourt, Rivers State, Nigeria

**Keywords:** Patent ductus arteriosus, tuberculosis, children, comorbidity, case report

## Abstract

Patent ductus arteriosus beyond the early neonatal period presents with failure to thrive, congestive cardiac failure, and recurrent pneumonia which is similar to the presentation of pulmonary tuberculosis. Both clinical conditions can coexist with significant adverse outcomes if not properly treated. This is a case of a 9-month-old female who had a hemodynamically significant patent ductus arteriosus (PDA). She had a surgical ligation of the PDA, and postoperative recovery was stalled by pulmonary tuberculosis which was initially missed as her post-operative symptoms were thought to have been from a post-op complication. She however progressively worsened till the diagnosis of pulmonary tuberculosis (PTB) was mading a suggestive chest X-ray finding. She was treated for PTB and made remarkable improvement with the resolution of the respiratory symptoms and adequate weight gain. A child with a symptomatic congenital cardiac defect in a TB endemic area can still have pulmonary tuberculosis which should not be missed. Also, the diagnosis of TB in children can be challenging due to laboratory tests that could have relatively poor yield when compared to adults. Hence, to avoid missing the diagnosis, a combination of clinical, laboratory, and regional epidemiology correlation is essential.

## Introduction

Patent ductus arteriosus (PDA) causes increased pulmonary blood flow which can lead to congestive heart failure, failure to thrive, and pulmonary vascular occlusive disease if untreated [1]. The treatment of PDA beyond the neonatal period is ductal occlusion via surgical ligation or insertion of a trans-catheter occlusion device. Post-PDA closure is followed by the resolution of cardiac and pulmonary symptoms with improved oxygen saturation and weight gain, postoperative complications are uncommon [2]. Pulmonary tuberculosis (TB) on the other hand is a common cause of childhood morbidity in TB-endemic regions [3] and the clinical presentation of pulmonary tuberculosis in children can be similar to symptoms seen in children with acyanotic congenital heart disease with the failure to thrive, respiratory distress, crepitation and chronicity of clinical symptoms. The presence of both morbidities in one patient has not been reported in literature and treatment outcomes can be negatively impacted if one or the order is neglected when managing patients. In children, a high index of suspicion is required to make a diagnosis of tuberculosis which could coexist with a cardiac pathology and complicate its clinical course even after the right treatment has been given. This is the case report of the clinical management challenges of a patient with both PDA and PTB.

## Patient and observation

**Patient information:** a 9-month-old female presented with a history of poor weight gain from birth, cough, and fever of 2 weeks, fast breathing of 1 week, and refusal to suck for one day. Weight loss was progressive despite adequate feeding. Cough was distressful, non-paroxysmal with no history of contact with an adult having a chronic cough. The fever was high-grade and continuous. Fast breathing was of insidious onset and had been progressive. She was unable to suckle from the breast for 24 hours prior to presentation despite her mothers´ concerted effort to breastfeed her. A review of systems revealed she had orthopnea, easy fatigability, excessive sweating, no cyanosis, no body swelling, and no seizures. She was referred to another hospital where she was admitted and had a blood transfusion without remarkable improvement. Prior to this, she had no previous hospital admission. Pregnancy, birth, and neonatal period were normal. Although her mother did not remember the birth weight. She was exclusively breastfed and commenced satisfactory complementary feeds. She was also adequately immunized and her development was within normal limits. She is the third of 3 children. Both parents were in their early thirties and from a low socio-economic class.

**Clinical findings:** at presentation, she had dysmorphic features with upward slanting of the palpebral fissures, low-set ears, and flattened nasal bridge. She was also severely pale, in severe respiratory distress with subcoastal and intercoastal recessions, flaring alae nasi and she was saturating at 85% in room air. Her weight was 6kg (44% of expected and > -3 Z-score), height was 72cm (0.8 Z-score). She was tachypneic with a respiratory rate of 60 cycles per minute and had widespread crepitation. She also had an active precordium, apex beat was displaced to the fifth left intercostal space lateral to the mid-clavicular line, she had tachycardia with a heart rate of 160bpm and a grade 3/6 continuous systolic murmur, maximal at the left lower sternal border and radiating to the armpit. Abdominal examination showed a reducible umbilical hernia and tender hepatomegaly with liver 4cm below the right subcoastal margin. An initial working diagnosis was severe bronchopneumonia in heart failure in a child with acyanotic congenital heart disease and chromosomal abnormality probably Downs syndrome, failure to thrive with severe malnutrition.

**Figure 1:**
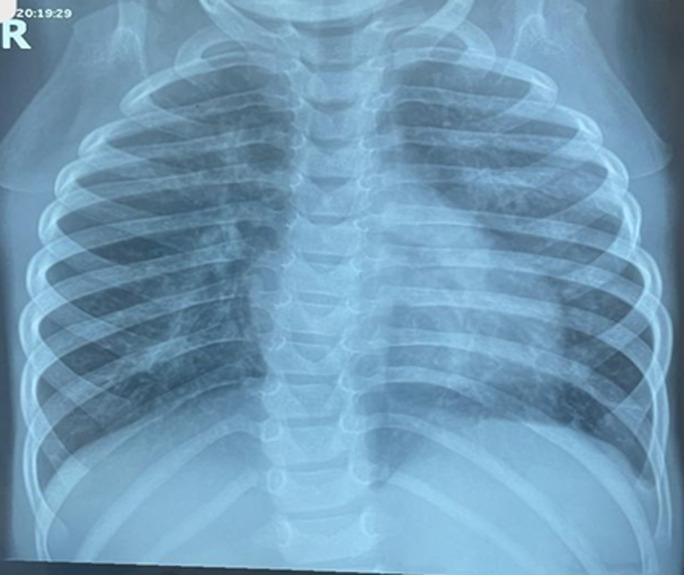
cardiogenic pulmonary edema showing perihilar mottled and fluffy opacities, cardiomegaly, and splaying of the carina

**Diagnostic assessment:** full blood count showed absolute leukocytosis with a white blood cell count of 22.7 x 109/L, and anemia with a packed cell volume of 28%, electrolytes urea, and creatinine showed hypokalemia of 2.4mmol/L, hypocalcemia with non-ionic calcium of 1.09mmol/L. The retroviral screening was seronegative. Chest X-ray (Figure 1), done showed: significant perihilar mottled and fluffy opacities involving all lung zones bilaterally, cardiomegaly with a cardiothoracic ratio of 61%, splaying of the carina suggestive of cardiogenic pulmonary edema. Electrocardiograph showed: sinus tachycardia, with a heart rate of 168bpm, right (rt) atrial enlargement, rt ventricle hypertrophy, and rt axis deviation. Echocardiography showed: 0.4cm PDA with continuous left to rt shunt at 74mmHg. The malaria parasite was positive.

**Figure 2:**
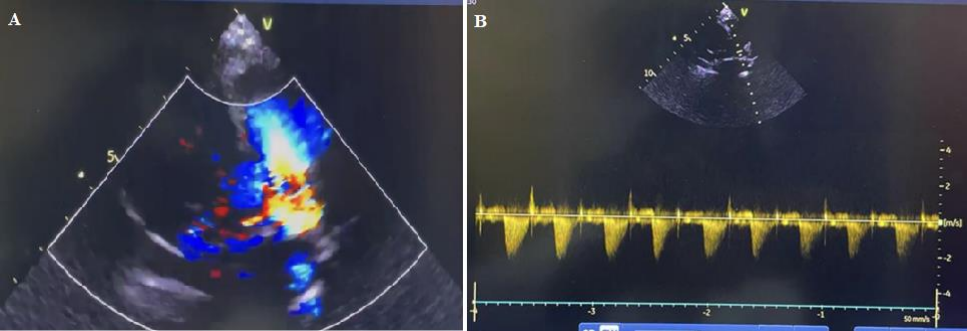
A) color doppler post-PDA ligation showing, no residual PDA flow with non-obstructive supravalvular narrowing; B) post-PDA ligation: good occlusion with no residual flow

**Therapeutic interventions:** she was, commenced on parenteral antibiotics, diuretics, intranasal oxygen, nutritional rehabilitation, vitamin D supplementation, antimalarial medications, and hematinic. She was stabilized, discharged, and referred for PDA ligation which she had after 6 months. The operation was successful as there was good PDA ligation with no residual flow on the echocardiogram (Figure 2 A,B).

**Figure 3:**
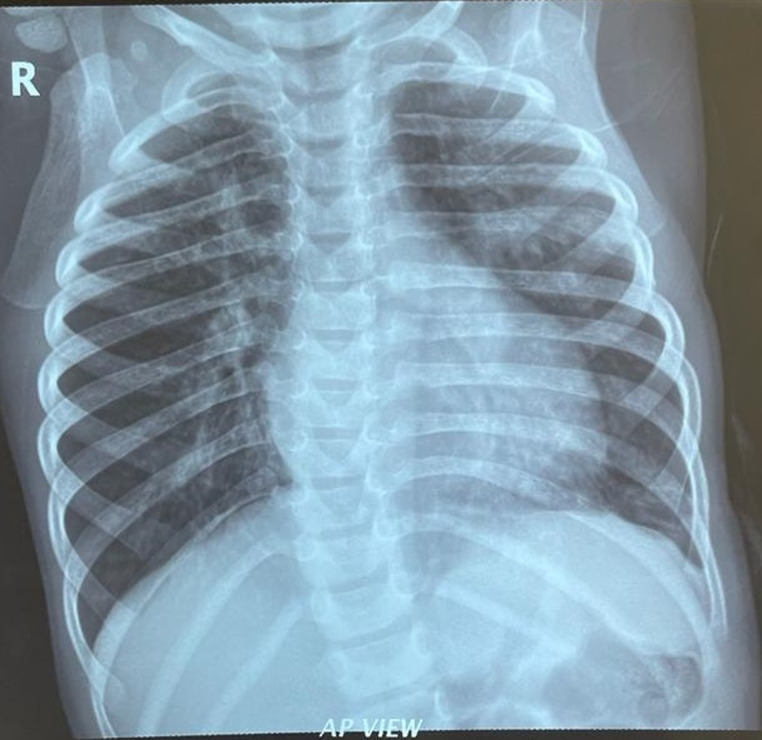
hilar and perihilar mottled opacities, normal cardiothymic silhouette

**Follow-up and outcome of interventions:** a month post-surgery she started coughing and though she had started gaining weight which was now 94% of expected, she had localized rhonchi in the left upper lung zone posteriorly, she was managed for early pneumonia with a possible localized lung obstruction as a post-PDA ligation complication. A chest X-ray was ordered which was not done although she was placed on antibiotics, and chest physiotherapy. She was managed for uncomplicated pneumonia two more times on an outpatient basis. The cough however progressively worsened over 3 months and she also developed a fever and started losing the previously gained weight. On subsequent examination, she was tachypneic, and had coarse crepitations, bilaterally. She also had tachycardia with a gallop rhythm and tender hepatomegaly. She was admitted for severe bronchopneumonia in heart failure to rule out PTB. Chest X-ray (Figure 3) showed bilateral perihilar mottled opacity. GeneXpert test showed mycobacteria tuberculosis was not detected. A diagnosis of PTB was made and she was commenced on anti-tuberculosis drugs with a good response. She has had no recurrence of pneumonia and started gaining weight adequately six months into the treatment.

**Patient perspective:** the mother was very satisfied and happy that her child has finally recovered.

**Informed consent:** a written informed consent was given by the mother to have her child´s case published.

## Discussion

This case highlights the reality of co-morbidity involving congenital and acquired conditions that can occur in pediatric practice and the need for a combination of clinical, laboratory, and regional epidemiology correlation in the diagnosis and appropriate management of pediatric illnesses. Our patient had dysmorphic features characteristic of Trisomy 21 with symptomatic patent ductus arteriosus, failure to thrive, and recurrent severe bronchopneumonia in heart failure which responded temporarily to appropriate antibiotic therapy, anti-heart failure regimen, and nutritional rehabilitation. She subsequently had surgical ligation of the PDA with significant improvement. However, the recurrence of severe bronchopneumonia and loss of previously gained weight led to an additional diagnosis of pulmonary tuberculosis which was supported by radiological features of hilar and peri-hilar lymphadenopathy on chest X-ray, although her GeneXpert test was negative. Anti-tuberculous drugs were commenced on clinical grounds to which she responded with no recurrence of repeat episodes of pneumonia.

Patent ductus arteriosus is a persistence of the communication between the pulmonary artery and the descending aorta. In fetal circulation, the ductus arteriosus serves as a partway by which oxygen-rich blood from the right ventricle bypasses the lungs due to its high pulmonary vascular resistance and supplies systemic circulation. After delivery, however, with the fall in pulmonary vascular resistance, blood should differentially pass into the lungs and away from the ductus resulting in a functional closure within 18 hours of birth and anatomic closure in 2-3 weeks [1]. The hemodynamic consequence of a persistent ductus flow includes an increase in left to right shunt from the aorta to the pulmonary artery with an increase in pulmonary blood flow and the development of pulmonary vascular occlusive disease which can ultimately be fatal. Clinical presentation is a recurrent chest infection, failure to thrive, and congestive heart failure as seen in our patient, the degree of symptomatology is determined by the volume of blood across the shunt. Prematurity, chromosomal abnormalities as seen in our patient, low birth weight, hypoxia, and genetic factors are associated with an increased risk of PDA [1].

Treatment of PDA includes medical closure with non-steroidal anti-inflammatory drugs within the first 14 days of life and medical management of congestive heart failure. Early surgical closure is indicated when there is a failure of medical closure and the presence of congestive heart failure. All PDA however, would need to be closed to prevent the risk of infective endocarditis. There are two types of PDA closure namely; transcatheter device and surgical closure. The choice of the type depends on the size of the PDA, the skill of the physician, and the resources available. Surgical ligation is the treatment of choice for large PDAs that require treatment in infants [1]. While transcatheter occlusion is the treatment of choice for PDAs in older children [2]. In resource-poor settings, poor financing and lack of skilled manpower limit the choice of use of catheter device occlusion. Our patient had surgical PDA closure. After a successful PDA closure, the signs of congestive heart failure should resolve, the PDA murmur should no more be audible and the patient should start gaining weight. Our patient seemed to have recovered post-surgery till a month after when respiratory symptoms and recurrent pneumonia warranted further investigation.

Although PDA surgical ligation is a relatively safe procedure with possible complications of injury to the recurrent laryngeal nerve, inadvertent occlusion of the left main bronchus, and ligation of the left pulmonary artery or aorta [4], our patient did not have any of these. However, the timing of symptomatic localized rhonchi in the left upper lung zone a month after PDA surgical closure made us think that the respiratory symptom was closely related to the surgical procedure. Hospital records of her immediate post-operative recovery events were also not available to our hospital on the patient´s return a month post-op. The importance of proper transfer of care with a detailed medical report cannot be over-emphasized for patients who have to travel away from their primary care provider, this information would usually help early decision-making in health care. Types of surgical PDA closure method range from the use of clips, ligation, and division, or ligation only, and certain complications could be method specific [4,5].

Laboratory diagnosis of TB in children is very challenging due to lack of sputum production, and poor bacterial load in the respiratory secretions because the bacilli usually remain confined to their perihilar lymph node without rupturing into the bronchus, hence, about 95% of TB cases are missed in children via the conventional diagnostic methods of acid-fast bacillus (AFB) in sputum [6]. GeneXpert has a sensitivity of 66% and specificity of 98% in diagnosing TB in children using sputum specimens [7], while from fecal specimens its sensitivity is 50% with 99% specificity [8]. Standard chest radiograph has a sensitivity of 38.8% and specificity of 74.4% in diagnosing children with PTB [9]. Diagnosis of TB is significantly improved when the interpretation of these laboratory findings is done in the context of the child´s clinical presentation as the presence of well-defined symptoms can classify a child into suspected or Probable tuberculosis. Suspected TB is made when two conditions are met, the first is a history of chronic illness with symptoms of cough with or without fever, weight loss or poor weight gain, fatigue, wheezing, or failing to make full recovery after measles or whooping cough while the second condition is the presence of one or more of the following; lymphadenopathies, malnutrition, pathologic chest signs, meningeal irritation, ascites, hepatomegaly or splenomegaly. Probable TB is when there is suspected TB in the presence of a positive tuberculin skin test, chest radiographic findings suggestive of TB, histologic findings of caseation necrosis in a biopsy specimen, or poor response to treatment after 2 weeks of antibiotics or good response to anti-tuberculous treatment [10]. Our patient met the criteria for probable TB. Although she did not do tuberculin skin testing (TST), her severely malnourished clinical state would have affected the result giving a false negative. Besides, TST would have prolonged the time it took to confirm the diagnosis by up to 48 to 72 hours when compared to the 24-hour turnaround time for the GeneXpert test which our patient did. There is also the added inconvenience of a needle prick with TST. Diagnosis of Tb in children is enhanced by a combination of epidemiologic, clinical, and radiologic findings and other laboratory tests.

Although PTB is a common cause of morbidity under-5 in our environment, co-morbidity with PDA post-ligation has not been documented. Her prompt response following the commencement of anti-Koch´s therapy underscores the need for a high index of suspicion in children who fail to respond to conventional treatment for specific morbidities. Our patient completed Tb treatment for 6 months with rifampicin, isoniazid, and pryrazinamie for 2 months followed by 4 months of isoniziazide and rifampicin during the maintenance phase. She has not had a recurrence of bronchopneumonia or other TB-defining symptoms 6 months after treatment and she is still being followed up.

## Conclusion

Patent ductus arteriosus (PDA) is an acyanotic congenital heart defect that can be treated with a good response. Pulmonary tuberculosis in children can also coexist with other comorbidities. A high index of suspicion is needed to make a diagnosis of PTB in the background of children with acyanotic congenital heart disease because of their similar presentations. Prompt diagnosis and treatment of both conditions yield good outcomes.
